# Challenges in Managing Pericardial Disease Related to Post Viral Syndrome After COVID-19 Infection

**DOI:** 10.7759/cureus.13461

**Published:** 2021-02-20

**Authors:** Zachary W Sollie, Shirisha R Vallepu, Cindrel Tharumia Jagadeesan, Lori C White, Vishnu Nagalapuram

**Affiliations:** 1 Internal Medicine, University of Alabama at Birmingham School of Medicine, Birmingham, USA; 2 Internal Medicine, University of Alabama at Birmingham, Montgomery Internal Medicine, Montgomery, USA; 3 Internal Medicine, St. Joseph's Hospital and Medical Center / Creighton University, Phoenix, USA

**Keywords:** post viral syndrome, pericardial disease, covid-19, viral pericarditis, cardiac tamponade

## Abstract

Although primarily a respiratory illness, coronavirus disease 2019 (COVID-19) has been associated with cardiac involvement with reported cases of myocardial ischemia, arrhythmia, myocarditis, pericarditis, and pericardial effusion leading to cardiac tamponade. Most cases of pericardial disease in this setting have been during the acute infection. Here, we present a patient who developed pericarditis leading to cardiac tamponade after the resolution of the acute COVID-19 infection. Her course of illness was further complicated by poor response to initial medical therapy with non-steroidal anti-inflammatory drugs (NSAIDs) and colchicine which could possibly be related to early exposure to steroids. It is often difficult to establish an underlying etiology for acute pericarditis. Similarly, in our case, although there is no definitive test to prove the causal relationship, this effusion is highly suspicious of being secondary to post viral sequelae after COVID-19 infection when considering the clinical course. It is important to consider pericardial disease as a late complication of COVID-19 even after apparent resolution of the acute infection and be mindful of the therapeutic challenges that we might face while managing such patients.

## Introduction

Coronavirus disease 2019 (COVID-19), a novel virus within the coronavirus family, was first described in December of 2019 [1]. While the primary concern of this virus is its potentially devastating pulmonary disease, it was quickly determined that there were numerous extra-pulmonary manifestations of this virus, including renal, gastrointestinal, liver, cardiac, mediastinal, neurologic, hematologic, vascular, cutaneous, reproductive, and ocular complications [2]. A wide variety of pathologies related to cardiac involvement in COVID-19 have been reported [3, 4]. In particular, the pericardial disease has been increasingly recognized to complicate the course of illness, given very little direction on management strategies. Acute pericarditis, myopericarditis, pericardial effusion, and cardiac tamponade have been reported so far but mostly in the setting of an acute infection with COVID-19 in conjunction with respiratory symptoms of the disease [5-8]. Only a few cases have been reported with cardiac manifestations as the sole presentation or as a late complication [9, 10]. Here, we present a patient who presented with pericardial effusion leading to tamponade as post-viral sequelae of COVID-19, which was further complicated by a protracted disease course.

## Case presentation

A 29-year-old previously healthy African American lady was diagnosed with COVID-19 in the middle of June 2020 when she had myalgia, fatigue, and headache following close contact with a friend who had a COVID-19 infection. She was given a course of steroid taper at an urgent care center. Given the mild nature of the disease, she required only supportive therapy at home along with self-isolation. After three weeks, she tested negative and was cleared from home quarantine. Three days after testing negative, she presented to the hospital with chest pain and shortness of breath. At that time, an electrocardiogram (EKG) showed sinus tachycardia, and an echocardiogram showed mild pericardial effusion of less than 1 cm. Significant labs upon presentation included negative troponins, erythrocyte sedimentation rate (ESR) of 74 mm per hour, C-reactive protein (CRP) of 3.39 mg/dL, COVID-19 immunoglobulin G of 68.9 AU/mL, anti-nuclear antibodies negative, and negative human immunodeficiency virus (Table 1). She was diagnosed with acute pericarditis and was discharged with a combination of colchicine and ibuprofen.

**Table 1:** Laboratory investigation ESR - erythrocyte sedimentation rate; CRP - C-reactive protein; ANA - anti-nuclear antibodies; WBC - white blood cells; RBC - red blood cells; PMN - polymorphonuclear leukocyte; LDH - lactate dehydrogenase; AFB - acid-fast bacilli; IgG - immunoglobulin G; COVID-19 - coronavirus disease 2019; HIV - human immunodeficiency virus

	Acute pericarditis presentation	Cardiac tamponade presentation	Lab reference values
ESR	74 m/hr	62 mm/hr	0-20 mm/hr
CRP	3.39 mg/dL	6.6 mg/dL	<0.8 mg/dL
Troponin	<2.5 ng/L	<2.5ng/dL	2.5-34.11 ng/L
ANA screen	Negative		
COVID-19 IgG	68.9 AU/mL		<1.4 AU/mL
HIV screen	Negative		
Pericardial fluid			
Appearance		Opaque	
Color		Bloody	
WBC		5,681/mm^3^	
RBC		163,000/mm^3^	
Manual monocytes		42%	
Manual PMN		56%	
Eosinophils		2%	
Protein		5.2 gm/dL	
LDH		511 mU/mL	
Albumin		3.3 gm/dL	
Bacterial culture		Negative	
AFB		Negative	

**Figure 1:**
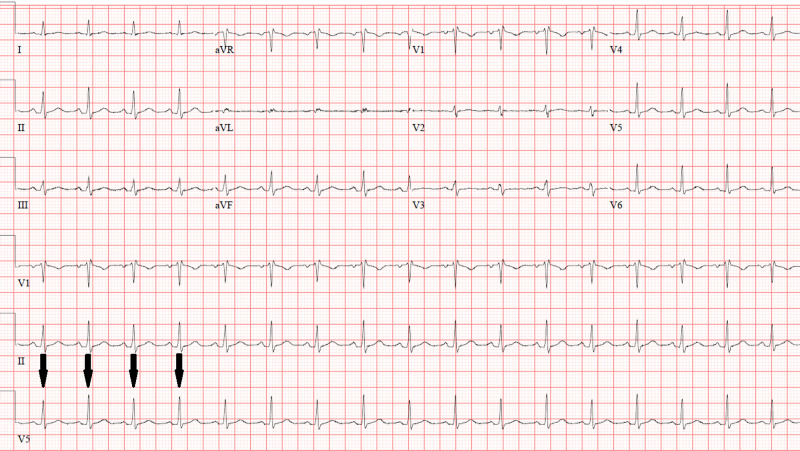
Electrocardiogram on return presentation demonstrating electric alternans

**Figure 2:**
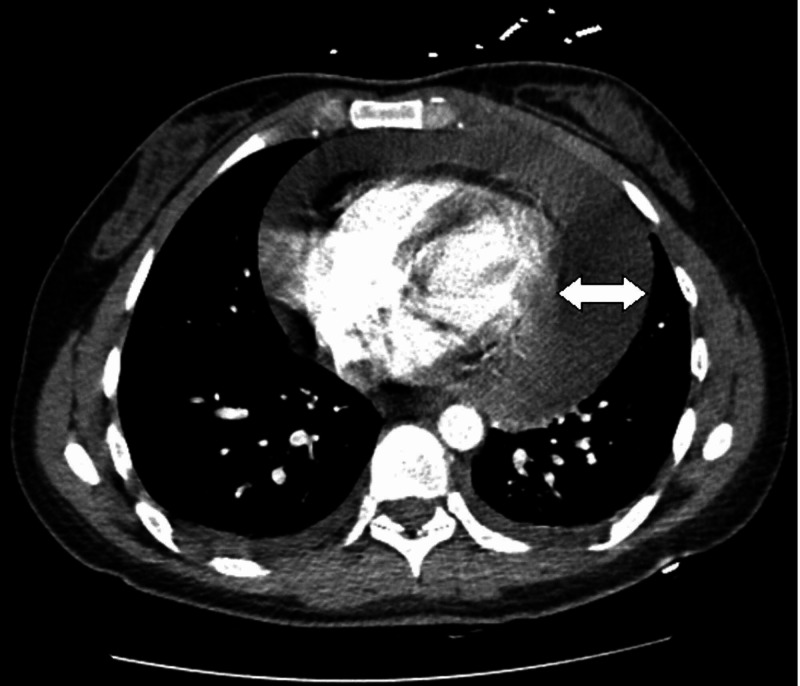
Axial view of the computed tomography angiogram of the chest revealing a large circumferential pericardial effusion

**Figure 3:**
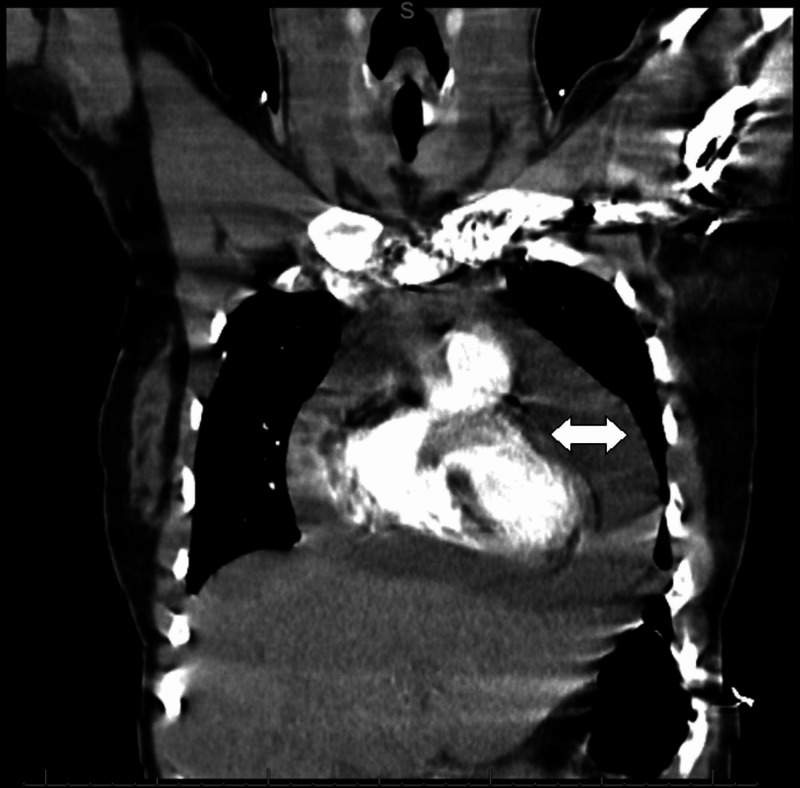
Coronal view of the computed tomography angiogram of the chest revealing a large circumferential pericardial effusion

**Figure 4:**
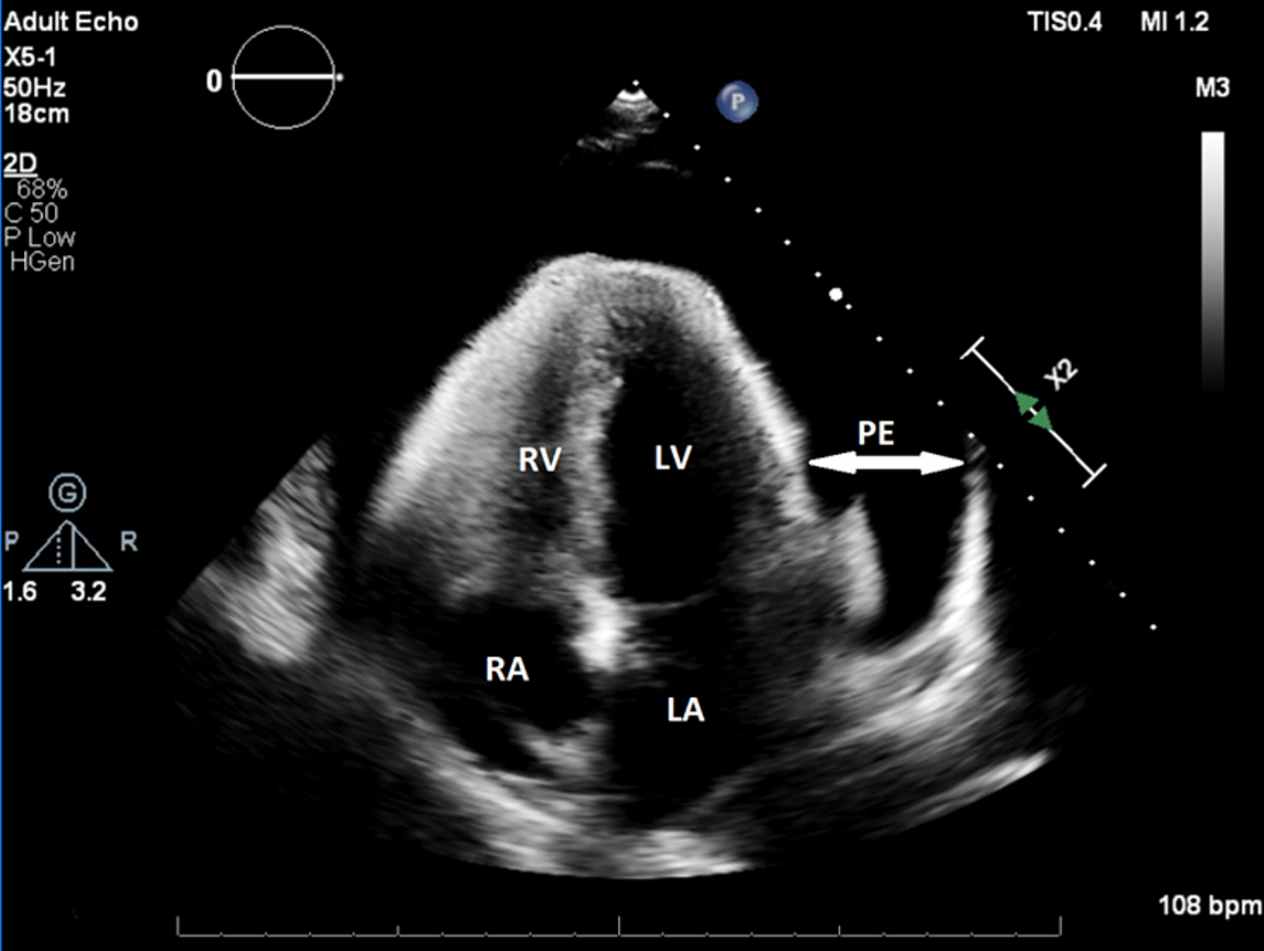
Apical four-chamber view of the 2D transthoracic echocardiogram revealing a large pericardial effusion LA - left atrium; RA - right atrium; LV - left ventricle; RV - right ventricle; PE - pericardial effusion

Five days following her initial discharge, she returned to the emergency department due to progressively worsening chest pain and exertional dyspnea. On presentation, her vital signs revealed a heart rate of 118 beats per minute and blood pressure of 111/85 mm Hg. Physical exam revealed a young woman in apparent distress, jugular venous pulse elevated to the mandible, and distant heart sounds with no rubs or murmurs. Pulsus paradoxus was noted upon further examination. Her EKG showed electrical alternans (Figure 1), and a computed tomography (CT) angiogram of the chest done to evaluate for pulmonary embolism revealed a large circumferential pericardial effusion (Figures 2-3). An echocardiogram revealed an increased effusion from her study seven days prior at greater than 3.5 cm and evidence of the diastolic collapse of the right ventricle (Figure 4). Upon recognizing the diagnosis, she underwent emergent pericardiocentesis, where 900 mL of serosanguinous fluid was drained. The pericardial fluid analysis revealed white blood cell count of 5,681/ mm^3^, red blood cell count of 163,000/mm^3^, protein of 5.2 gm/dL, lactate dehydrogenase of 511 mU/mL, negative bacterial cultures, and negative acid-fast bacilli (Table 1). Testing for COVID- 19 in the pericardial fluid was not available at our center. A drain was left in place for approximately 24 hours to reassess accumulation. Following a 24-hour echocardiogram revealed minimal effusion, the drain was removed, and her symptoms improved.

Considering no response to ibuprofen, we managed her with a high dose of 325 mg aspirin three times daily with a slow taper along with colchicine twice daily for three months. Adjunctive therapy consisted of pain management with opioids and incentive spirometry for atelectasis. Prior to discharge, she received a follow-up echocardiogram, which showed a stable effusion with a measurement of less than 1 cm. During the hospital stay, surgical consultation for consideration of the pericardial window was obtained. Through shared decision-making, it was determined to reserve this procedure for recurrence or chronic course. Her symptoms persisted at her one-week follow-up, for which she was switched from taking aspirin to prednisone along with colchicine. Symptoms resolved with this regimen adjustment, and it was continued for three weeks followed by a three-month taper, at which point she didn’t have any pericardial effusion on echocardiogram.

## Discussion

Since the beginning of the COVID-19 pandemic, pericardial disease related to COVID- 19 has been reported increasingly. The range of presentations with this pathology varies, including pericarditis as the sole primary presentation of COVID-19 infection, pericarditis in conjunction with respiratory symptoms, pericardial effusion with or without cardiac tamponade, pericarditis with features of myocarditis, and pericarditis in a pediatric patient [5-11]. At least two out of the following four are needed for the diagnosis of acute pericarditis: 1) chest pain; 2) pericardial rub; 3) EKG changes, and 4) new or worsening pericardial effusion [12]. This should hold true even in the context of COVID-19, whether acute or as a post-viral syndrome. No specific biomarker is available to diagnose pericarditis. However, inflammatory markers like ESR, CRP, leukocyte count, and imaging modalities, including chest roentgenogram, CT scan, and echocardiogram, are often utilized to diagnose and monitor response to treatment. Cardiac specific biomarker - troponin - can be used to assess myocardial involvement. Advanced imaging like cardiac magnetic resonance should be reserved for ambiguous cases [13]. COVID-19 testing on the pericardial fluid is neither available widely nor validated. However, COVID-19 has been demonstrated in the pericardial fluid using reverse transcriptase-polymerase chain reaction and electron microscopy [14, 15]. Since management would not change depending on this, it is reasonable to avoid these investigations, which are often laborious and expensive. The yield of pericardial fluid analysis is often poor in establishing a viral etiology and can often be irrelevant clinically since most cases can be treated empirically without identifying a specific viral etiology [16]. A limitation to our case was the lack of further investigation for common viral etiologies that can lead to pericardial disease. While many other common viruses are known to have pericardial sequelae, the clinical picture of known recent COVID-19 infection was enough to raise suspicion. Furthermore, management of viral-related pericarditis likely would not have otherwise changed. 

Medications that have been employed so far in pericardial disease related to COVID-19 include colchicine, NSAIDs including aspirin, steroids, and intravenous immunoglobulins (IVIG) [6, 7, 17]. Colchicine has been used alone or in combination with NSAIDs successfully [5, 7, 10]. Li et al. reported a case of a hemodynamically unstable patient with COVID-19 myopericarditis who responded well to IVIG and methylprednisolone [17]. At the beginning of the pandemic, there was some safety concern related to NSAIDs use in COVID-19 [18]. However, more recent data suggest no increased risk of severe adverse outcomes related to this [19]. Early use of steroids in non-COVID-19 related pericarditis has been linked to increased recurrence and poor long-term outcomes [20]. In our patient, we propose that her poor response to ibuprofen and aspirin, along with her protracted course, is likely related to early steroid exposure prescribed originally for her mild COVID-19 infection. This question should further be explored, but it is unlikely that we would have an answer soon, given how uncommon pericardial disease is in this setting. Hence, it is not unreasonable to continue to choose NSAIDs along with colchicine and avoid steroids for treating pericardial disease related to COVID-19 infection. Invasive interventions include pericardiocentesis more often than pericardiectomy [6-11].

Overall, acute pericarditis should be recognized in the setting of COVID-19, just like other viral infections. This can be related to the acute infection itself or as post-viral sequelae. Evaluation and diagnosis are largely the same. However, treatment modalities should be carefully selected. Diagnostic pericardiocentesis can be avoided if there are no high-risk features. A trial of colchicine can be considered in pericardial disease related to COVID-19. Anecdotally, a course of steroid taper is often prescribed by providers for mild COVID-19 infection without a clear benefit. In order to provide the most benefit to the patient when steroids are needed, we feel it may be helpful to avoid steroids early in the course of the infection unless benefits related to hypoxia outweigh other risks. Other medications like NSAIDs, intravenous immunoglobulins, etc., should be individualized. Therapeutic pericardiocentesis is necessary for patients with cardiac tamponade. Pericardiectomy should be reserved for recurrent or chronic pericarditis as usual.

## Conclusions

Pericardial disease as a cardiac manifestation of COVID-19, both in acute infection and as post-viral sequelae, should be promptly recognized. Steroids should be avoided in mild COVID-19 and in acute pericarditis since it could worsen the long-term outcomes of pericarditis itself. Colchicine can be considered in all patients with pericardial disease, and other therapies could be individualized. 
